# A matching‐adjusted indirect comparison of survival outcomes with pirtobrutinib (BRUIN) versus standard of care (SCHOLAR‐2) in relapsed/refractory mantle cell lymphoma previously treated with a covalent Bruton tyrosine kinase inhibitor

**DOI:** 10.1111/bjh.70237

**Published:** 2025-11-17

**Authors:** Georg Heß, Toby A. Eyre, Min‐Hua Jen, Jiewen Zhang, Benjamin Goebel, Sarang Abhyankar, Martin Dreyling

**Affiliations:** ^1^ Department of Hematology and Medical Oncology University Medical School of the Johannes Gutenberg‐University Mainz Germany; ^2^ Department of Haematology, Cancer and Haematology Centre Oxford University Hospitals NHS Foundation Trust Oxford UK; ^3^ Eli Lilly and Company Ltd. Indianapolis Indiana USA; ^4^ TechData Service Company King of Prussia Pennsylvania USA; ^5^ Medizinische Klinik III, LMU Klinikum Munich Germany

**Keywords:** covalent BTK inhibitor, non‐covalent BTK inhibitor, pirtobrutinib, relapsed/refractory mantle cell lymphoma

To the Editor,

Mantle cell lymphoma (MCL) is generally associated with a continuous pattern of relapse following first‐line treatment. In the relapsed/refractory (R/R) setting, covalent Bruton tyrosine kinase inhibitors (cBTKi; ibrutinib, acalabrutinib, zanubrutinib) have become standard of care (SOC).^1^, ^2^, ^3^ Although cBTKi are associated with high overall response rates (ORR), MCL continues to progress.^4^ cBTKi discontinuation due to toxicity/intolerance has also been an issue^5^ and alternative therapeutic options remain relatively limited.^1^, ^2^

SCHOLAR‐2, a recently published retrospective chart review study, reported survival outcomes of 240 patients with R/R MCL in Europe who were treated with cBTKi‐based therapy from 2012 to 2018 and had disease progression while on a cBTKi or discontinued cBTKi therapy due to intolerance.^6^ SCHOLAR‐2 was performed prior to the routine availability of chimeric antigen receptor T‐cell (CAR‐T) treatment, which is now considered standard in cases of cBTKi resistance or intolerance. Median overall survival (OS) from cBTKi initiation was 14.6 months (95% confidence interval [CI] 11.6–20.0). 149/240 patients (62%) received post‐cBTKi treatment. For this group, median OS from first post‐cBTKi therapy initiation was 9.7 months (95% CI 6.3–12.7). These results provide a suitable benchmark for the survival of real‐world patients with R/R MCL receiving SOC therapy after cBTKi failure (in a landscape excluding CAR‐T therapy).

Pirtobrutinib is an orally available, highly selective, non‐covalent BTKi which has recently received United States Food and Drug Administration and European Medicines Agency approval for the treatment of R/R MCL. Based on the results of the BRUIN trial, pirtobrutinib is approved for use after ≥2 lines of systemic therapy, including prior cBTKi in the United States, and after previous treatment with a cBTKi in the European Union.^7^, ^8^ In BRUIN, pirtobrutinib was safe and effective regardless of previous cBTKi resistance or intolerance,^9^ and updates indicate that pirtobrutinib continues to demonstrate durable efficacy, a high ORR (49.3%) and a beneficial safety profile in heavily pretreated patients with R/R MCL and prior cBTKi therapy.^10^

We therefore sought to explore the OS of pirtobrutinib versus SOC (as per SCHOLAR‐2) for patients with R/R MCL previously treated with a cBTKi. We conducted a matching‐adjusted indirect comparison (MAIC) using individual patient‐level data from the BRUIN primary analysis set (PAS, prespecified primary efficacy cohort of 90 patients with measurable disease, but not central nervous system involvement, who had received a prior cBTKi) and data from patients who received treatment after a cBTKi in SCHOLAR‐2 (SCHOLAR‐2 subcohort that received post‐cBTKi treatment, *n* = 149/240).^6^, ^11^

Clinical/disease characteristics and prior treatment details were used to re‐weight patients in the BRUIN PAS to match those within the published SCHOLAR‐2 subcohort with post‐cBTKi treatment. Matching was performed according to a previously published methodology involving reweighting patients in the BRUIN PAS by their odds of having been enrolled in a trial without available individual patient data (SCHOLAR‐2).^12^ Details of the methodology used are provided in the Supporting Information.

Baseline demographic and clinical characteristics of patients in the BRUIN PAS versus the SCHOLAR‐2 subcohort are provided in Table S1. The data cut‐off date for BRUIN PAS was 29 July 2022 (minimum survival follow‐up: 6 months). Median survival follow‐up was 23.5 months for the BRUIN PAS and 27.3 months for the SCHOLAR‐2 subcohort. Overall, BRUIN had a greater proportion of male patients (80.0% vs. 72.5%), similar median patient age (70 vs. 71 years), similar proportions of stages III and IV combined (87.5% vs. 88.3%) and a smaller proportion with blastoid or pleomorphic histology versus the SCHOLAR‐2 subcohort (22.2% vs. 41.3%). Median number of prior treatment lines was 3 for both studies. The SCHOLAR‐2 subcohort included more patients with ≥3 lines of therapy (62.4% vs. 54.4% in BRUIN) and previous autologous haematopoietic stem cell transplant (32.2% vs. 18.9%). SCHOLAR‐2 showed no clearly dominant post‐cBTKi treatment regimen; 52.3% of patients received chemotherapy (± anti‐CD20 monoclonal antibody), most frequently bendamustine plus rituximab (15.8%), and targeted therapies were delivered to 45.0% of patients (± anti‐CD20 antibodies), most frequently lenalidomide‐containing regimens (17.4%). Median OS from initiation of post‐cBTKi treatment in BRUIN (pirtobrutinib) was 23.5 months (95% CI: 15.9 to not reached)^11^ versus 9.7 months (95% CI: 6.3–12.7) in SCHOLAR‐2 (SOC).^6^ In both unadjusted and adjusted (MAIC) analyses, pirtobrutinib demonstrated an OS advantage versus SOC in SCHOLAR‐2 (unadjusted hazard ratio [HR] = 0.473, 95% CI: 0.324–0.689, *p* < 0.001; adjusted HR = 0.497, 95% CI: 0.327–0.756, *p* = 0.001) (Figure 1).

**FIGURE 1:**
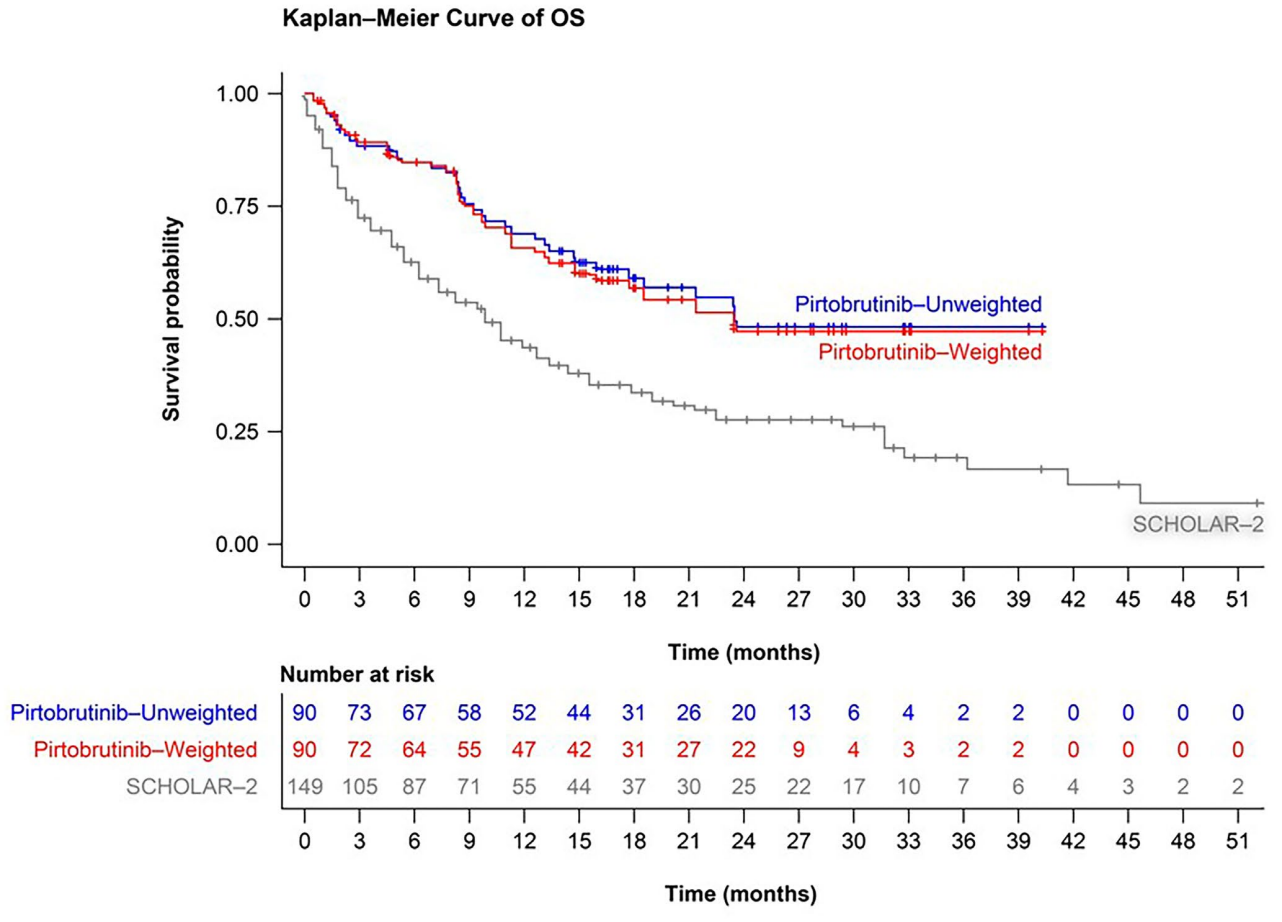
Overall survival (OS) probability for patients in: (i) the BRUIN PAS without applying weights; (ii) the BRUIN PAS after applying weights to match the SCHOLAR‐2 subcohort; and (iii) the SCHOLAR‐2 subcohort. PAS, primary analysis set.

In the BRUIN PAS,^11^ with a median OS follow‐up of 23.5 months, ORR was 56.7% (95% CI: 45.8%–67.1%) and the median duration of response was 17.6 months (95% CI: 7.3–27.2 months).^11^, ^13^ The data cut‐off date of 29 July 2022 allowed that the majority (>90%) of patients responding to pirtobrutinib (i.e. achieving a complete response [CR, *n* = 17/90, 18.9%] or partial response [PR, *n* = 34/90, 37.8%]) be followed up for ≥9 months from the onset of the initial response. Because prognosis was poor in non‐responders to pirtobrutinib, progression‐free survival (PFS) and OS were also determined in patients responding to pirtobrutinib (*n* = 51/90). In these 51 patients, the median PFS was 19.5 months (95% CI: 9.1–19.2), which was associated with prolonged OS (median not reached and 18‐month OS rate 80.4%) (Figure 2).

**FIGURE 2:**
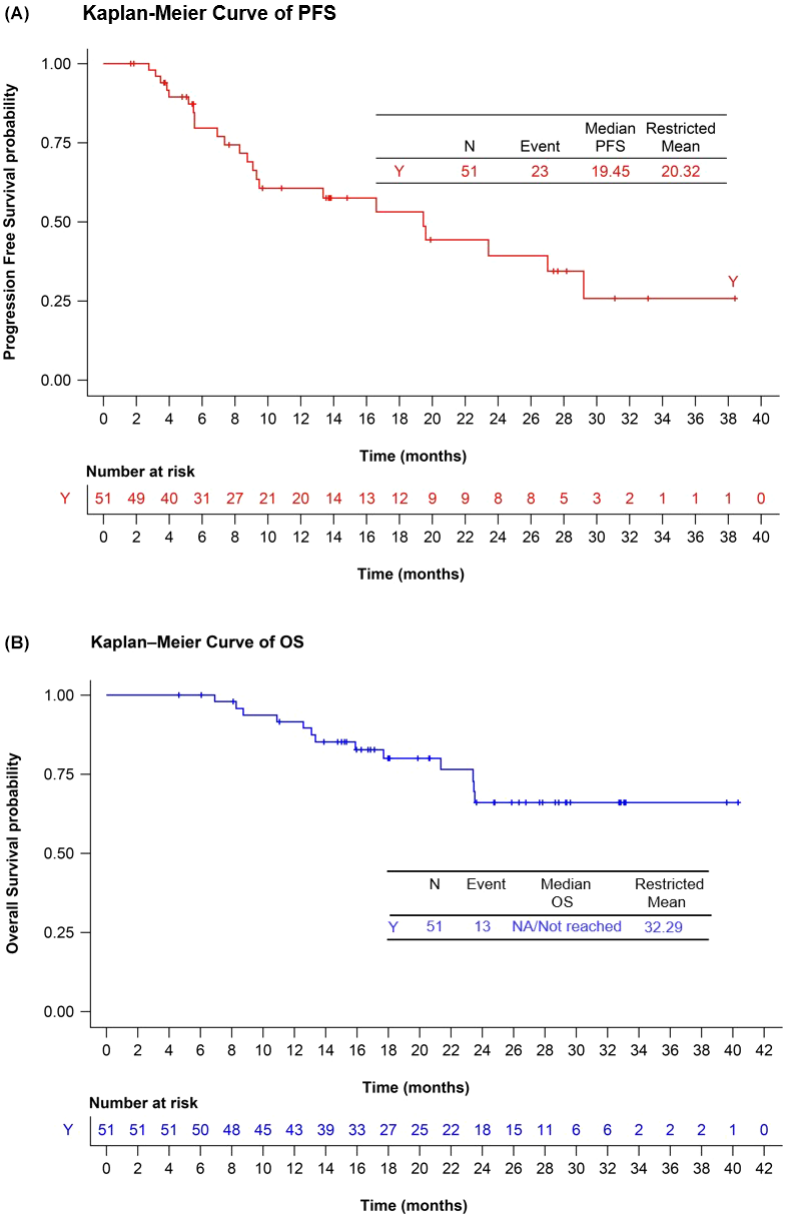
(A) Progression‐free survival (PFS) and (B) overall survival (OS) for patients with relapsed/refractory mantle cell lymphoma (R/R MCL) who responded to pirtobrutinib in the BRUIN PAS (*n* = 51).^13^ PAS, primary analysis set. [Correction added on 29 November 2025, after first online publication: Figure 2 has been corrected.]

MAIC is limited in that matching baseline characteristics and/or effect modifiers may not remove all confounders, and other differences in trial populations, including the presence/absence of TP53 gene mutation, indicators of poor prognosis such as POD24, designs and conduct, may influence treatment effects. Nevertheless, despite the heterogeneity of the two studies (BRUIN includes a prospective clinical trial cohort and SCHOLAR‐2 includes a retrospective non‐selected cohort) and missing data that prevented better matching of the two cohorts, findings may suggest a possible OS benefit with pirtobrutinib versus SOC for patients with R/R MCL after cBTKi treatment. That BRUIN and SCHOLAR‐2 collected data on patients treated across different eras may also have influenced survival outcomes due to relative access to novel therapies. CAR‐T therapy represents another treatment option in this setting, also showing a clinical advantage versus SOC in an indirect comparison^14^; however, it is associated with barriers, such as patient factors, accessibility issues, clinically meaningful turnaround time from screening to administration, therapy resistance, considerable toxicity and significant cost.^1^, ^15^ Thus, non‐covalent BTK inhibition with pirtobrutinib addresses a significant unmet clinical need, especially for those achieving disease control.

## AUTHOR CONTRIBUTIONS

Georg Heß contributed to the conception of the work, the design of the work, the acquisition of data for the work, the analysis of data for the work and to the interpretation of data for the work. Toby A. Eyre contributed to the acquisition of data for the work, the analysis of data for the work and the interpretation of data for the work. Min‐Hua Jen contributed to the conception of the work, the design of the work, the acquisition of data for the work, the analysis of data for the work and to the interpretation of data for the work. Benjamin Goebel contributed to the conception of the work. Sarang Abhyankar contributed to the conception of the work and to the interpretation of data for the work. Jiewen Zhang contributed to the analysis of data for the work and to the interpretation of data for the work. Martin Dreyling contributed to the conception of the work, the design of the work, the acquisition of data for the work, the analysis of data for the work and to the interpretation of data for the work. All authors contributed to the critical revision of the work for important intellectual content and approved the work to be published or presented.

## FUNDING INFORMATION

The publication of this article was funded by Eli Lilly and Company.

## CONFLICT OF INTEREST STATEMENT

Georg Heß has received grants or contracts from Kite, a Gilead Company, Incyte, Janssen, Morphosys, Pfizer, Roche and AbbVie; has received consulting fees from AbbVie, ADC‐Therapeutics, AstraZeneca, BMS, Genmab, Kite, a Gilead Company, Incyte, Janssen, Miltenyi, Novartis, Roche and Lilly; has received payment or honoraria for lectures, presentations, speakers' bureaus, manuscript writing or educational events from AbbVie, AstraZeneca, Beigene, BMS, Genmab, Kite, a Gilead Company, Incyte, Janssen, Lilly and Roche; has received support for attending meetings and/or travel from Kite, a Gilead Company and Janssen; and has participated on a Data Safety Monitoring Board or Advisory Board for Miltenyi. Toby A. Eyre has received research funding from AstraZeneca and Beigene; has received consulting fees from Abbvie, AstraZeneca, Autolus, Beigene, Incyte, Janssen, Kite, Lilly and Roche; has received payments or honoraria for lectures, presentations, speakers' bureaus, manuscript writing or educational events from Abbvie, AstraZeneca, Autolus, Beigene, Incyte, Janssen, Kite, Lilly and Roche; has participated on a Data Safety Monitoring Board/Advisory Board with Galapagos and a Trial Steering Committee with AstraZeneca, Beigene, BMS, Lilly and Roche. Min‐Hua Jen, Benjamin Goebel and Sarang Abhyankar are employees of and hold shares in Eli Lilly and Company. Jiewen Zhang has no conflicting interests to declare. Martin Dreyling has received institutional support for clinical trials from AbbVie, Bayer, Celgene/BMS, Kite, a Gilead Company, Janssen and Roche; has received payment or honoraria for lectures, presentations, speakers' bureaus, manuscript writing or educational events from Astra Zeneca, Beigene, Kite, a Gilead Company, Janssen, Lilly, Novartis and Roche; has received support for attending meetings and/or travel from Janssen and Roche; and has participated on a Data Safety Monitoring Board or Advisory Board for AbbVie, AstraZeneca, Beigene, Celgene/BMS, Kite, a Gilead Company; Janssen, Lilly/Loxo, Novartis, Roche.

## ETHICS APPROVAL STATEMENT

This article is based on previously conducted studies and does not contain any new studies with human participants or animals performed by any of the authors.

## PATIENT CONSENT STATEMENT

For the matching‐adjusted indirect comparison, no patients were treated in the current analyses. For the BRUIN trial, institutional review boards or independent ethics committees overseeing each site approved the protocol. The study was performed according to the Declaration of Helsinki, Good Clinical Practice guidelines and local laws. All patients provided written informed consent.

## PERMISSION TO REPRODUCE MATERIAL FROM OTHER SOURCES

Permission is not required for the material presented in this disclosure.

## CLINICAL TRIAL REGISTRATION (INCLUDING TRIAL NUMBER)

As this was a matching‐adjusted indirect comparison, no clinical trial registration was required. The BRUIN trial is registered with ClinicalTrials.gov, NCT03740529.

## Supporting information


Data S1.

## Data Availability

The datasets generated and/or analysed during the current study are available from the corresponding author on reasonable request.

## References

[1] Jain P , Wang ML . Mantle cell lymphoma in 2022 – a comprehensive update on molecular pathogenesis, risk stratification, clinical approach, and current and novel treatments. Am J Hematol. 2022;97(5):638–656. 10.1002/ajh.26523 35266562

[2] Eyre TA , Bishton MJ , McCulloch R , O'Reilly M , Sanderson R , Menon G , et al. Diagnosis and management of mantle cell lymphoma: a British Society for Haematology guideline. Br J Haematol. 2024;204(1):108–126. 10.1111/bjh.19131 37880821

[3] Silkenstedt E , Dreyling M . Treatment of relapsed/refractory MCL. Blood. 2025;145(7):673–682. 10.1182/blood.2023022353 39059015

[4] Martin P , Maddocks K , Leonard JP , Ruan J , Goy A , Wagner‐Johnston N , et al. Postibrutinib outcomes in patients with mantle cell lymphoma. Blood. 2016;127(12):1559–1563. 10.1182/blood-2015-10-673145 26764355

[5] Coutre SE , Byrd JC , Hillmen P , Barrientos JC , Barr PM , Devereux S , et al. Long‐term safety of single‐agent ibrutinib in patients with chronic lymphocytic leukemia in 3 pivotal studies. Blood Adv. 2019;3(12):1799–1807. 10.1182/bloodadvances.2018028761 31196847 PMC6595265

[6] Hess G , Dreyling M , Oberic L , Gine E , Zinzani PL , Linton K , et al. Real‐world experience among patients with relapsed/refractory mantle cell lymphoma after Bruton tyrosine kinase inhibitor failure in Europe: the SCHOLAR‐2 retrospective chart review study. Br J Haematol. 2023;202(4):749–759. 10.1111/bjh.18519 36257914 PMC10812379

[7] Jaypirca [Summary of Product Characteristics] [Internet]. Available from: https://www.ema.europa.eu/en/documents/product‐information/jaypirca‐epar‐product‐information_en.pdf

[8] Jaypirca [Prescribing Information] [Internet]. Indianapolis, IN: Eli Lilly and Company; 2023. Available from: https://pi.lilly.com/us/jaypirca‐uspi.pdf

[9] Mato AR , Shah NN , Jurczak W , Cheah CY , Pagel JM , Woyach JA , et al. Pirtobrutinib in relapsed or refractory B‐cell malignancies (BRUIN): a phase 1/2 study. Lancet. 2021;397(10277):892–901. 10.1016/S0140-6736(21)00224-5 33676628 PMC11758240

[10] Cohen JB , Shah NN , Jurczak W , Zinzani PL , Cheah CY , Eyre TA , et al. 981 Pirtobrutinib in relapsed/refractory (R/R) mantle cell lymphoma (MCL) patients with prior cBTKi: safety and efficacy including high‐risk subgroup analyses from the phase 1/2 BRUIN study. American Society of Hematology Annual Meeting & Exposition 2023. https://ash.confex.com/ash/2023/webprogram/Paper181627.html

[11] Jurczak W , Zinzani PL , Eyre TA , Cheah CY , Ujjani CS , Izutsu K , et al. Pirtobrutinib in covalent BTK‐inhibitor pre‐treated mantle cell lymphoma: updated results and subgroup analysis from the phase 1/2 BRUIN study with >3 years follow‐up from start of enrollment. Hemasphere. 2023;7(Suppl):e45636b5. 10.1097/01.HS9.0000971244.45636.b5

[12] Phillippo DM , Ades AE , Dias S , Palmer S , Abrams KR , Welton N , et al. NICE DSU Technical Support Document 18: Methods for Population‐Adjusted Indirect Comparisons in Submission to NICE [Internet]. December 2016. Available from: https://www.sheffield.ac.uk/nice‐dsu/tsds/population‐adjusted

[13] European Medicines Agency . Orphan Maintenance Assessment Report. Jaypirca (pirtobrutinib). Treatment of Mantle Cell Lymphoma. EU/3/21/2450 [Internet]. 30 October 2023. Available from: https://www.ema.europa.eu/en/documents/orphan‐maintenance‐report/jaypirca‐epar‐orphan‐maintenance‐assessment‐report‐initial‐authorisation_en.pdf

[14] Hess G , Dreyling M , Oberic L , Gine E , Zinzani PL , Linton K , et al. Indirect treatment comparison of brexucabtagene autoleucel (ZUMA‐2) versus standard of care (SCHOLAR‐2) in relapsed/refractory mantle cell lymphoma. Leuk Lymphoma. 2024;65(1):14–25. 10.1080/10428194.2023.2268228 37840282

[15] Newcomb R , Jacobson C . Chimeric antigen receptor T cells for B‐cell lymphoma. Cancer J. 2021;27(2):107–111. 10.1097/PPO.0000000000000509 33750069

