# Supplementary material for: A matching‐adjusted indirect comparison of survival outcomes with pirtobrutinib (BRUIN) versus standard of care (SCHOLAR‐2) in relapsed/refractory mantle cell lymphoma previously treated with a covalent Bruton tyrosine kinase inhibitor
Source: Br J Haematol. 2025 Nov 17;208(1):312–6. doi: 10.1111/bjh.70237 (PMC12819100; doi:10.1111/bjh.70237)
Supplement: Supplementary file 1 — Data S1. [file BJH-208-312-s001.docx]

**Supplemental file**

**Statistical methods**

To investigate the overall survival of pirtobrutinib^1^ (based on the phase I/II BRUIN study^2^) versus standard of care (as represented by the SCHOLAR-2 study^3^) for patients with relapsing/remitting mantle cell lymphoma (MCL) previously treated with a covalent Bruton tyrosine kinase inhibitor (cBTKi), a matching-adjusted indirect comparison (MAIC) was performed.

As patient-level data were available from BRUIN and summary data from SCHOLAR-2, the methodology for MAIC presented in the National Institute for Health and Care Excellence (NICE) Decision Support Unit (DSU) Technical Support Document (TSD) 18 guidelines^4^ were used to make comparisons between the trials.

A weighted unanchored MAIC^5^ was conducted using data from the 149 patients who received treatment post-cBTKi in SCHOLAR-2 rather than the full population (i.e., it excludes 91 patients who did not receive post-cBTKi treatment) to align with BRUIN and the BRUIN PAS population using R statistics. Matching was performed according to a previously published methodology (reweighting patients in the BRUIN PAS by their odds of having been enrolled in the trial without available individual patient data [IPD] – SCHOLAR-2).^4^ A logistic regression model was estimated using the method of moments so that the weight for each individual patient was equal to the patient’s estimated odds (propensity) of being in the BRUIN PAS versus the SCHOLAR-2 sub-cohort.^4-6^ Distribution of the applied weights was inspected for potential extreme values, which could be indicative of poor overlap between the study populations in the distributions of patient characteristics.^7^ Survival outcomes were compared using Cox regression and log-rank tests.

The primary analysis used an informed covariate approach, whereby the covariates used in the reweighting exercise were limited to those with literature supporting their prognostic value. A previous MAIC of brexucabtagene autoleucel (ZUMA-2) versus standard of care (SCHOLAR-2) in people with relapsed/refractory (R/R) MCL included population adjustment for imbalances in prognostic factors between the ZUMA-2 and SCHOLAR-2 study populations.^8^ This study was evaluated to identify potential baseline characteristics for the adjustments in the current study. Hess et al^8^ identified the following baseline characteristics as prognostic factors relevant to patients with R/R MCL: number of previous lines of treatment, previous autologous stem cell transplant, duration on previous cBTKi therapy, response to previous cBTKi therapy (overall response rate; ORR), MCL International Prognostic Index (MIPI) or simplified MIPI (sMIPI) (low, intermediate, high), morphologic variants (blastoid), Ki67 (≥ 30%, ≥ 50%), disease staging (stage 3, stage 4), previous cBTKi therapy (ibrutinib), sex (male), extranodal disease (lymphoma excluding bone marrow and splenic involvement), and bone marrow involvement.^9^ Individual components of the MIPI and sMIPI were also noted as prognostic factors (age, lactate dehydrogenase [upper limit normal], white blood cells count, Eastern Cooperative Oncology Group performance status), but were not considered for the MAIC due to inclusion of the MIPI composite risk scores.^9^ Additionally, tumour protein p53 mutation, bulky disease, and primary refractory disease status were considered as potential prognostic factors but were excluded from the final list because of inconsistent reporting across comparator studies.^9^

MIPI was not included in the current analysis because of the high proportion of missing data. Among the components of MIPI, age was the only variable with complete data reported in SCHOLAR-2; therefore, it was initially considered for adjustment in our analysis. However, given that the mean (standard deviation) age was comparable between the two studies (69.4 (8.76) years vs 70.9 (9.45) years), we ultimately decided against its inclusion in the final analysis. The baseline covariates previous lines of treatment, previous autologous stem cell transplant, previous cBTKi ORR, disease stage, sex, extranodal disease, and bone marrow involvement were therefore used to re-weight the patients in the BRUIN PAS to match those in the SCHOLAR-2 sub-cohort. These variables comprise the available data from both studies that have been identified as prognostic.

**References**

1. Wang ML, Jurczak W, Zinzani PL, et al. Pirtobrutinib in Covalent Bruton Tyrosine Kinase Inhibitor Pretreated Mantle-Cell Lymphoma. *J Clin Oncol*. 2023;41(24):3988-3997. doi:10.1200/JCO.23.00562
2. Jurczak W, Zinzani PL, Eyre TA, et al. Pirtobrutinib in covalent BTK-inhibitor pre-treated mantle cell lymphoma: updated results and subgroup analysis from the phase 1/2 BRUIN study with >3 years follow-up from start of enrollment. *Hemasphere*. 2023;7(Suppl ):e45636b5. doi: 10.1097/01.HS9.0000971244.45636.b5
3. Hess G, Dreyling M, Oberic L, et al. Real-world experience among patients with relapsed/refractory mantle cell lymphoma after Bruton tyrosine kinase inhibitor failure in Europe: The SCHOLAR-2 retrospective chart review study. *Br J Haematol*. 2023;202(4):749-759. doi:10.1111/bjh.18519
4. Phillippo DM, Ades AE, Dias S, Palmer S, Abrams KR, Welton NR. NICE DSU technical support document 18: Methods for population-adjusted indirect comparisons in submission to NICE. December 2016. [https://www.sheffield.ac.uk/nice-dsu/tsds/population-adjusted. Accessed 02 August 2024](https://www.sheffield.ac.uk/nice-dsu/tsds/population-adjusted.%20Accessed%2002%20August%202024)
5. Signorovitch JE, Sikirica V, Erder MH, et al. Matching-adjusted indirect comparisons: a new tool for timely comparative effectiveness research. *Value Health*. 2012;15(6):940-947. doi:10.1016/j.jval.2012.05.004
6. Signorovitch JE, Wu EQ, Yu AP, et al. Comparative effectiveness without head-to-head trials: a method for matching-adjusted indirect comparisons applied to psoriasis treatment with adalimumab or etanercept. *Pharmacoeconomics*. 2010;28(10):935-945. doi:10.2165/11538370-000000000-00000
7. Jiang Y, Ni W. Performance of unanchored matching-adjusted indirect comparison (MAIC) for the evidence synthesis of single-arm trials with time-to-event outcomes. *BMC Med Res Methodol*. 2020;20(1):241. Published 2020 Sep 29. doi:10.1186/s12874-020-01124-6
8. Hess G, Dreyling M, Oberic L, et al. Indirect treatment comparison of brexucabtagene autoleucel (ZUMA-2) versus standard of care (SCHOLAR-2) in relapsed/refractory mantle cell lymphoma. *Leuk Lymphoma*. 2024;65(1):14-25. doi:10.1080/10428194.2023.2268228
9. National Institute for Health and Care Excellence. Brexucabtagene autoleucel for treating relapsed or refractory mantle cell lymphoma. Technology appraisal guidance TA677. 24 February 2021. Single Technology Appraisal. Autologous anti-CD19-transduced CD3+ cells for treating relapsed or refractory mantle cell lymphoma [ID1313]. Committee Papers. <https://www.nice.org.uk/guidance/ta677/evidence/committee-papers-pdf-9016513021> [last accessed 27 September 2024]

| Baseline characteristics | | SCHOLAR-2 sub-cohort | BRUIN (PAS)  [unadjusted] | BRUIN (PAS)  [adjusted] |
| --- | --- | --- | --- | --- |
| **Sample size (n)** | | 149 | 90 | ESS = 59.2 |
| ***Characteristics used for re-weighting*** | |  |  |  |
| **Previous lines of treatment** | |  |  |  |
| 1-2 | | 56/149 (37.6%) | 41/90 (45.6%) | 38% |
| 3 | | 44/149 (29.5%) | 18/90 (20.0%) | 30% |
| 4+ | | 49/149 (32.9%) | 31/90 (34.4%) | 32% |
| **Previous autoHCT** | | 48/149 (32.2%) | 17/90 (18.9%) | 32% |
| **Previous cBTKi ORR** | | 46/133 (34.6%) | 41/90 (45.6%)^a,b^ | 35% |
| **Disease stage III+** | | 98/111 (88.3%) | 77/88 (87.5%) | 88% |
| **Male** | | 108/149 (72.5%) | 72/90 (80.0%) | 72% |
| **Extranodal disease** | | 29/100 (29.0%) | 35/90 (38.9%) | 29% |
| **Bone marrow involvement** | | 45/100 (45.0%) | 46/90 (51.1%) | 45% |
| ***Additional characteristics*** |  |  |  |  |
| **sMIPI** |  |  |  |  |
| Low | | 3/62 (4.8%) | 20/90 (22.2%) |  |
| Intermediate | | 14/62 (22.6%) | 50/90 (55.6%) |  |
| High risk | | 45/62 (72.6%) | 20/90 (22.2%) |  |
| Missing | | 87/149 (58.4%) | -- |  |
| **Histology at initial diagnosis** | |  |  |  |
| Classic/leukemic (BRUIN) or non-blastoid or non-pleomorphic (SCHOLAR-2) | | 27/46 (58.7%) | 70/90 (77.8%) |  |
| Blastoid | | 17/46 (37.0%) | 8/90 (8.9%) |  |
| Pleomorphic | | 2/46 (4.3%) | 12/90 (13.3%) |  |
| Missing | | 103/149 (69.1%) | -- |  |
| **Median age, years (range)** | | 71 (43–91) | 70 (46–87) |  |
| **Disease stage** | |  |  |  |
| **I** | | 5/111 (4.5%) | 4/88 (4.58%) |  |
| **II** | | 8/111 (7.2%) | 7/88 (7.9%) |  |
| **III** | | 20/111 (18.0%) | 8/88 (9.1%) |  |
| **IV** | | 78/111 (70.3%) | 69/88 (78.4%) |  |
|  | | 98/111 (88.3%) | 77/88 (87.5%) |  |
| **ECOG PS** | |  |  |  |
| **0** | | 29/105 (27.6%) | 61/90 (67.8%) |  |
| **1** | | 39/105 (37.1%) | 28/90 (31.1%) |  |
| **2** | | -- | 1/90 (1.1%) |  |
| **≥ 2** | | 37/105 (35.2%) | 1/90 (1.1%) |  |
| **Reason for discontinuation of most recent cBTKi** | |  |  |  |
| Disease progression | | 127/149 (85.2%) | 73/90 (81.1%) |  |
| Toxicity | | 22/149 (14.8%) | 12/90 (13.3%) |  |
| Other | | -- | 5/90 (5.6%) |  |
| **Previous alloHCT** | | 3/149 (2.0%) | 4/90 (4.4%) |  |

Data reported as n (%) unless specified otherwise for characteristics reported for both trials. BRUIN reported normalised LDH whereas SCHOLAR-2 reported median LDH; BRIUN did not report on Ki67 Proliferation Index, presence of B symptoms or splenic involvement, and SCHOLAR-2 did not report on white cell counts or previous cBTKi response other than ORR.

alloHCT: allogeneic hematopoietic stem cell transplant; autoHCT: autologous hematopoietic stem cell transplant; cBTKi: covalent Bruton’s tyrosine kinase inhibitor; ECOG: Eastern Cooperative Oncology Group; ESS: effective sample size; LDH: lactate dehydrogenase; n: number of participants in the specified category; ORR: overall response rate; PAS: primary analysis set; PS: performance status; sMIPI: simplified mantle cell lymphoma international prognostic index.

^a^ BRUIN response data are based on the most recent cBTKi received

^b^ Calculated including patients with unknown data in the denominator

Supplemental Table 1. Baseline characteristics of patients in the BRUIN PAS vs the SCHOLAR-2 sub-cohort
